# The Newcastle satisfaction with nursing scales in a Mexican Oncology Hospital

**DOI:** 10.4314/ahs.v21i1.10

**Published:** 2021-03

**Authors:** Cecilia Rodríguez-Herrera, José de Jesús López-Jiménez, Azucena del Toro-Valero, Nora Magdalena Torres-Carrillo, Norma Torres-Carrillo, Carlos Alberto Godínez-Peña, Ana Cecilia Méndez-Magaña, Melva Guadalupe Herrera-Godina, Ana Lilia Fletes-Rayas

**Affiliations:** 1 Instituto Jalisciense de Cancerología; 2 Instituto Mexicano del Seguro Social, Centro de Investigación Biomédica de Occidente, División de Medicina Molecular; 3 Universidad de Guadalajara, Centro Universitario de Ciencias de la Salud, Departamento de Disciplinas Filosófico Metodológico e Instrumentales; Departamento de Morfología; 4 Universidad de Guadalajara, Centro Universitario de Ciencias de la Salud, Departamento de Microbiología y Patología; 5 Universidad de Guadalajara, Centro Universitario de Ciencias de la Salud, Departamento de Fisiología; 6 Universidad de Guadalajara, Centro Universitario de Ciencias de la Salud, Departamento de Salud Pública; 7 Universidad de Guadalajara, Centro Universitario de Ciencias de la Salud, Departamento de Psicología Aplicada; 8 Universidad de Guadalajara, Centro Universitario de Ciencias de la Salud, Departamento de Enfermería Clínica Aplicada

**Keywords:** Spanish satisfaction tool, hospital, patient satisfaction, oncology nursing, health care evaluation mechanisms

## Abstract

**Objectives:**

The principal aim of this study was to identify whether the Newcastle Satisfaction with Nursing Scales (NSNS) could be used on cancer patients.

**Methods:**

This was a descriptive, cross-sectional study carried out on cancer patients (n = 298).

**Results:**

We found that a majority of cancer patients were around 50 years old (hospitalized patients [HP]: 49.5 ± 14.9; chemotherapy outpatients [COP]: 49.4 ± 12.7), were female (HP: 74%; COP: 63.5%), and had received education at least up to elementary level (HP: 70%; COP: 80%). Breast cancer was the principal type of cancer (>34%) in both groups (HP and COP). The groups were comparable in age, sex distribution, place of origin, educational qualification, and type of cancer. Among HP, the experience and satisfaction scales of the NSNS showed good internal consistency (n = 235, α >0.9, r > 0.7), while among COP, only the satisfaction scale showed good internal consistency (n = 62, α = 1.00). Most patients' perceptions (level of satisfaction) of hospitalization and chemotherapy services were positive (98% and 97%, respectively).

**Conclusion:**

An NSNS instrument specifically designed for ambulatory care cancer patients is necessary for it to be useful in assessing cancer patients' perception of nursing care. This will help improve the quality of care in Mexico.

The presence of cancer by itself could modify the patients' satisfaction level. Further large-scale studies are required to investigate the patients' perceptions of nursing care using the NSNS on different cancer patient groups.

## Introduction

Patient satisfaction is defined as the subjective evaluation (cognitive and emotional responses) that results from the interaction between a patient's expectations with respect to nursing care (ideal) and her/his perceptions of the actual nursing care^1^. Patient satisfaction is also defined as a combination of experiences, expectations, and needs to be perceived^2^. Patients' opinions and subjective perceptions of the care they have received are regarded as important for quality improvement as they provide knowledge about the quality of health services^3^,^4^.

The quality of health services in Mexico is evaluated by a set of health quality indicators called “Sistema Nacional de Indicadores de Calidad en Salud” (INDICAS). The INDICAS is used to monitor and compare the quality of medical units across the country through nursing supervisors to accurately assess the technical procedures of the nurses under their charge. However, this system neglects the patients' subjective perceptions of the received care. INDICAS quality assessment system belongs to “Sistema Integral de Calidad en Salud” (SICALIDAD). SICALIDAD considers the patients' opinions of their treatment and attention given by medical personnel^5^. Unfortunately, this system contains generalized questions focusing on the relationship with “medical and nursing personnel,” and it does not comprehensively assess patients' perceptions of nurses and nursing care.

The Newcastle Satisfaction with Nursing Scales (NSNS) is regarded as an accurate assessment of patients' perceptions of nursing care. It is used to assess the views of users of health services in order to guide the development and progression of quality care in nursing units^6^.

Brazil was the first Latin American country to assess patients' perception of care using the NSNS7. Several other countries have used the NSNS as well, including Ethiopia, Turkey, Poland, Italy, Malaysia, the UK, and Canada^6^,^8^–^15^. In the Americas, there is still little information on care perception and satisfaction among patients using the NSNS questionnaire^10^,^16^. Furthermore, the NSNS has been used to assess patient satisfaction across diverse areas of nursing care and specialties (e.g., intensive care, medical and surgical nursing), and for different patient types (e.g., adolescents and adults). However, the NSNS has been applied neither to cancer patients nor to patients in Mexico. In Guadalajara city (Jalisco state), public hospitals provide health services to a large numbers of patients with cancer. In a public oncology hospital, the data shows that 127,462 medical consultations were granted between 2013 and 2018.

The aim of this study was to identify whether the NSNS could be used on Mexican cancer patients belonging to a public oncology hospital.

## Methods

This was a descriptive cross-sectional study; carried out using the NSNS version modified by Dorigan et al.^7^ This questionnaire has previously been translated and validated in a Brazilian population (patients from a public teaching hospital).

We used a sample of 298 patients receiving chemotherapy and care services at a single oncology public university hospital in Jalisco state, Mexico. Medical records were acquired for all patients. According to the mode of chemotherapy drug delivery, the population surveyed was divided into two groups: hospitalized patients (HP [n = 235]), whose stay lasted more than two days at the hospital, and chemotherapy outpatients (COP [n = 63]), whose stay lasted between 1–3 hours. The NSNS was translated from English to Spanish and tropicalized by two independent experts. During NSNS application, nurses were present to address patients' doubts in order to maintain instrument validity and reliability (Experiences of Nursing Care Scale [Cronbach´s α= 0.86], Satisfaction with Nursing Care Scale [Cronbach´s α=0.97]^16^.

### Ethical considerations

Written informed consent was obtained from all patients prior to the study. All procedures were approved by the institutional review board (number 014/16).

### Data analysis

The NSNS is rated on a seven-point Likert scale (modified by Dorigan et al.^7^) (Table 1) and consists of two separate scales: the Experiences of Nursing Care Scale (“A”) and the Satisfaction with Nursing Care Scale (“B”). The “A” scale evaluated the experience of the nursing care as perceived by the patients and contained 26 items (eleven items were negatively worded and therefore were reverse scored). The “B” scale evaluated patient satisfaction with how the nursing staff carried out nursing interventions and care. The NSNS was applied without changes for the HP group. However, as the outpatient chemotherapy service is provided only during the morning and afternoon shifts, all items referring to the night shift were removed for the COP group. This resulted in five items being eliminated—four from the “A” scale (“The nursing team turned the lights off too late at night,” “The nursing team explained what they were going to do to me before they did it,” “The nursing team used to go away and forget what patients had asked for,” and “The nursing team did not seem to know what each other were doing”) and one from the “B” scale (“The amount the nursing team knew about your care”).

**Table 1:** Internal consistency of the Newcastle Satisfaction with Nursing Scales for two hospital service areas and score values by service

	**Experience Scale items**		**Satisfaction Scale**		**α (r) Values**		

	**Cumulative score expressed by X, SD (Min-Max)**				A Items		B Items
	
**Hospitalization** **group (n=234)**	58 ± 1.2 (46 - 60)		99.1 ± 0.3 (85.7 - 100)		0.94 (0.75)		0.94 (0.91)
**Outpatient** **chemotherapy group** **(n= 62)**	64 ± 0.4 (62.9 - 65.2)		99.3 ± 0.2 (98 - 100)		0.58 (0.66)		1.000

	**Hospitalization group**		**Outpatient** **chemotherapy group**		**Cut off** **points**		

**Level of satisfaction,** **TOTAL A+B.** **X, SD (Min-Max), n**	75 ± 1.2 (66 - 77), n = 234		79.4 ± 0.1 (79.2 - 80), n = 62				
**Level of Satisfaction,** **n (%). Via** **demarcation** **threshold formula**	Fully satisfied = 230 (98%)		Fully satisfied = 60 (97%)		72 †		
	Not Fully satisfied = 4 (2%)		Not Fully satisfied = 2 (3%)		80 ‡		

**Ordinal Scale NSNS instrument**							

	**Disagree** **Completely**	**Disagree** **a lot**	**Disagree** **a Little**	**Neither** **Agree** **nor**	**Agree** **a little**	**Agree** **a lot**	**Agree** **completely**
**Decoding, qualitative** **ordinal scale** §	1	2	3	4	5	6	7
	^††^6	^††^5	^††^4	^††^3	^††^2	^††^1	^††^0

**X**= Average, **Min**= Minimum, **Max**= Maximum, **SD**= Standard Deviation

α = Cronbach's alpha

§ upper (Items) and down [positive and ^††^ negative questions (inversely proportional score)]

† Cut off point calculated using the demarcation threshold formula in Hospitalization service

‡ Cut of point calculated using the demarcation threshold formula in Outpatient chemotherapy service

The analysis was performed using SPSS (Statistical Package for the Social Sciences) v. 25 for Windows (IBM, Armonk, NY, USA). The internal consistency of the NSNS was confirmed via the Cronbach's α coefficient and Pearson's correlation coefficient r (good reliability was considered when the Cronbach's α was ≥ 0.70 and the r was ≥ 0.60)^17^.

Patient satisfaction was assessed by classifying participants into two groups: fully and not fully satisfied, using a cutoff point calculated via the demarcation threshold formula: [(total highest score – total lowest score)/2] + total lowest score^8^. As shown in Table 1, the cutoff point calculated by this formula for the HP group was 72; therefore, participants with scores of less than 72 were considered not fully satisfied and those with scores greater than 72 were considered as fully satisfied. For the COP group, the cutoff point was 79.6.

## Results

Patient characteristics are listed in Table 2. The groups were comparable in mean age, sex distribution, birthplace, educational qualification, and type of cancer (p = 0.05 – 1) but differing in marital status (p = 0.00). The mean age of patients was close to 50 years; most of them were females and had received education at least up to elementary level. Complete data are presented in Table 2.

**Table 2:** Demographic characteristics of cancer patients

		Hospitalization service (n = 235)	Outpatient chemotherapy service (n = 63)	*p*
Age (years), x̄ ± SD (range)		49.5 ± 14.9 (19–83)	49.4 ± 12.7 (27–77)	0.97 *

Sex distribution, n (%)	Female	174 (74)	40 (63.5)	0.12 **
	Male	61 (26)	23 (36.5)	

Marital status, n (%)	Married	157 (66.8)	48 (76.2)	**0.00** **
	Single	52 (22.1)	6 (9.8)	
	Widowed	26 (11.1)	2 (3.2)	
	Divorced	0	7 (11.1)	

State of Mexico, n (%)	Jalisco	197 (84)	53 (84)	1.00 **
	Others	38 (16)	10 (16)	

Education, n (%)	Illiterate	35 (15)	6 (10)	0.29 **
	Primary school	166 (70)	51 (80)	
	High school †	25 (11)	6 (10)	
	University	9 (4)	0	

Cancers by Body Location/System, n (%)	Breast	97 (41.3)	22 (34.9)	0.05 **
	Gynecologic	47 (20)	8 (12.7)	
	Digestive/Gastrointestinal	39 (16.6)	13 (20.6)	
	Hematologic/Blood	13 (5.5)	11 (17.5)	
	Musculoskeletal	11 (4.7)	0	
	Genitourinary	10 (4.3)	6 (9.5)	
	Head and Neck	8 (3.4)	2 (3.2)	
	Skin	4 (1.7)	1 (1.6)	
	Neurologic	3 (1.3)	0	
	Other	3 (1.3)	0	

† High school in the Mexican Educational System is divided into two categories. In the present report, these two categories were merged to form one.

* Student's *t*-test

** Fisher's Exact test

Using the demarcation threshold formula, the total score (experience scale “A” + satisfaction scale “B”) for the HP group was near to the maximum value (corresponding to fully satisfied, as shown in Table 1). The same was true for the COP group. Factor analysis techniques, including the Bartlett and Kaiser-Meyer-Olkin test, were not performed due to maximum score being achieved for the majority of positive items. This behaved like a forced-choice test and produced results that were like ipsative data.

Table 1 shows the Cronbach's α and correlation coefficients used to validate the NSNS applied to patients, contrasting between paired and non-paired items. Items in “A” and “B” were analyzed for both services. Only the “A” scale in the COP group did not show good internal consistency (α = 0.58 and r = 0.66) (Table 1).

Table 1 shows the mean item scores of the two scales broken down by patient service. Interestingly, the mean scores for the “A” scale were rather low for both patient groups owing to the negatively worded items, which had relatively high scores before being reversed. Using the demarcation threshold formula, the mean scores of the “B” scale indicated that patients' perceptions were resoundingly positive (near to the maximum score) for both groups. The total NSNS scores for both patient groups are also shown in Table 1. According to the above cutoffs, more than 96% of participants were fully satisfied with the hospitalization and outpatient chemotherapy services (Table 1).

## Discussion

The INDICAS and SICALIDAD are government programs focused on evaluating the quality of health services across Mexico. Both systems have limitations that delay the fulfillment of governmental objectives. The INDICAS system has a limited coverage (participating units do not exceed half of the total in Mexico); in addition, the participation of private institutions is nil^18^. The patients' perception of nursing care is evaluated only in terms of the care received through the dichotomous response to 11 questions that do not comprehensively evaluate the patients' perception of nursing staff and nursing care. On the other hand, NSNS is focused particularly on patients' perceptions about the nursing service.

Concerning the SICALIDAD system, quality managers who are responsible for filling and analyzing data devote their time to various functions, not all of which are necessarily related to the operation of SICALIDAD. A relevant aspect is that the evaluation and reporting of results in INDICAS and SICALIDAD are carried out by personnel belonging to the evaluated institution, which prevents timely monitoring of quality and reliability^19^.

Notably, the NSNS scale has been used widely across Europe^6^,^9^,^11^,^13^, but in the Americas, it only has been applied in Canada and Brazil^7^,^10^, and it has not, until now, been used to assess cancer patients.

In assessing the reliability of the NSNS in both patient services, we found that the Cronbach's α and correlation coefficient of the “A” scale were insufficient for the COP group (α = 0.58, r = 0.6612) (Table 1). This might be because the COP group has a short stay in the service and the NSNS was not specifically designed for ambulatory care.

In the hospitalization area, by contrast, the Cronbach's α was high (Table 1). Greater homogeneity and internal consistency were observed in the responses. This indicates that the NSNS is well suited and easily understood by these patients, similar to Dorigan et al.'s findings^16^. With respect to the level of satisfaction with nursing care, 96% of cancer patients were fully satisfied (see Table 1). Furthermore, in both services, the mean score on the experience and satisfaction scales was close to 60 and 90, respectively (Table 1). In other countries, fewer percentage of patients were satisfied with nursing care^8^,^12^,^13^.

Regarding the causes of annoyance in patients in this study, the following experiences were rated most negatively: “The nursing team favored some patients over others,” “The nursing team made me do things before I was ready,” “The nursing team let things get on top of them,” “The nursing team used to go away and forget what patients had asked for,” and “The nurses did not seem to know what the other members of the nursing staff were doing.” Most of the items (> 95%) in the hospitalization and chemotherapy services were scored with the highest values (rated most negatively) without finding a modal factor. In one descriptive study, it was found that the sleep disturbances factor was the major cause of annoyance^13^. Our results were not in accordance with Uğraş et al.'s findings^13^, in that we did not find a principal annoyance factor. With respect to the high negative score for one NSNS scale item, several studies^8^,^16^,^20^,^21^ reported similar results to Uğras et al.^13^. Interestingly, Gutysz-Wojnicka et al.9 reported that in university hospitals, the total satisfaction score was lower in comparison with provincial and district hospitals^9^. The hospital analyzed in this study was a university hospital, and the overall rate of full satisfaction was close to 95%.

It is currently recognized that satisfaction can be affected by various factors—i.e., older people, females, and less educated patients tend to assign high scores^20^–^22^. In the present study, most of the patients presented a high level of satisfaction; however, cancer patients had some capable factors to influencing their high scores, including sex, age, and level of education. It is also likely that the presence of cancer by itself could influence the assignment of high scores.

As noted above, the scores for the COP group were overly high (relative to the cutoff point calculated using the demarcation threshold formula), perhaps due to their short stay. On the other hand, the “A” scale scores were comparatively low, which could be because the NSNS was not designed for use in a public hospital wherein nurses' attention is influenced by a diverse range of factors, such as the short time allocated to care for each patient.

To the best of our knowledge, this is the first study to evaluate patient satisfaction in Mexico and to be applied to cancer patients using the NSNS. The analyzed hospital primarily treats a vulnerable population, as it is a specialized public hospital. Therefore, the questionnaire should be modified according to the hospital, the service, and the characteristics of the patients—i.e., the results of the present study may not represent reality for other hospitals, which is a potential limitation of the present study.

## Conclusion

The NSNS instrument is a useful tool for improving the quality of Mexican oncological health services and must be modified according to the hospital and service needs. The questionnaire showed good internal consistency and objectivity, especially in cancer patients who are hospitalized for longer intervals, and could be applied to other hospitals in Mexico. An NSNS instrument specifically designed for ambulatory care cancer patients is necessary. The presence of cancer could modify the level of patient satisfaction. Further largescale studies are required to investigate patients' perceptions of nursing care using the NSNS across the Americas, and on different patient groups.
